# AI-driven wastewater management through comparative analysis of feature selection techniques and predictive models

**DOI:** 10.1038/s41598-025-07124-0

**Published:** 2025-07-14

**Authors:** Faruk Dikmen, Ahmet Demir, Bestami Özkaya, Muhammad Owais Raza, Jawad Rasheed, Tunc Asuroglu, Shtwai Alsubai

**Affiliations:** 1 Department of Environmental Engineering, Yildiz Technical University, 34220 Istanbul, Turkey; 2 Department of Civil Engineering, Istinye University, 34396 Istanbul, Turkey; 3 Department of Computer Engineering, Istanbul Sabahattin Zaim University, 34303 Istanbul, Turkey; 4 Department of Software Engineering, Istanbul Nisantasi University, 34398 Istanbul, Turkey; 5 Applied Science Research Center, Applied Science Private University, Amman, Jordan; 6 Faculty of Medicine and Health Technology, Tampere University, 33720 Tampere, Finland; 7 VTT Technical Research Centre of Finland, 33101 Tampere, Finland; 8 Department of Computer Science, Prince Sattam Bin Abdulaziz University, 11942 Al-Kharj, Saudi Arabia

**Keywords:** Machine learning, Feature selection, Environmental engineering, Waste water treatment plan, Artificial intelligence, Computer science, Environmental sciences

## Abstract

The integration of artificial intelligence (AI) in wastewater treatment management offers a promising approach to optimizing effluent quality predictions and enhancing operational efficiency. This study evaluates the performance of machine learning models in predicting key wastewater effluent parameters Chemical Oxygen Demand (COD), Biochemical Oxygen Demand (BOD), Total Suspended Solids (TSS), Total Effluent Nitrogen and Total Effluent Phosphorus. Three feature selection techniques were applied: SelectKBest, Mutual Information, and Recursive Feature Elimination (RFE) using Random Forest to identify the most significant predictors. The study leveraged ensemble learning models, including XGBoost, Random Forest, Gradient Boosting, and LightGBM, and compared them with Decision Tree models. The results demonstrate that effluent volatile suspended solids (VSS) consistently held the highest predictive importance across all feature selection methods. Ensemble models significantly outperformed Decision Trees, with Gradient Boosting achieving the best predictive accuracy for TSS and total nitrogen (Mean Absolute Error (MAE): 3.667 $R^2$: 97.53), XGBoost excelling in COD prediction with MAE and $R^2$ of 6.251 and 83. 41%, respectively, and XGBoost showing superior performance for BOD (MAE: 1.589 $R^2$:79.64%). LightGBM yielded the highest precision in predicting total phosphate with MAE and a $R^2$ score of 0.230 and 28. 68%, respectively. Decision tree models consistently underperformed, exhibiting the highest error rates. These findings highlight the potential of AI-driven approaches in wastewater management to improve decision-making, regulatory compliance, and resource efficiency. However, limitations such as operational irregularities and seasonal variations remain challenges for further refinement.

## Introduction

The existence of life without water on Earth cannot be mentioned. Our civilization needs water for personal consumption and industrial purposes. 71% of the world’s surface consists of water, but 0.3% can be used. Our water needs are provided from fresh water resources such as ground water, rivers, and lakes^1^. Our world is a closed system in terms of our natural resources. There is now at erentry from an extra terrestrial system to our world. Therefore, water is a limited resource. The water that formed in the process of world formation billions of years ago is used today. Water is tasteless, odorless, and colorless. It is a chemically good solvent. It also provides the minerals we need for life from water because of this feature^2^. However, due to the same feature, it dissolves metals, toxic chemicals, pesticides, and harmful compounds used in industries^3^. After this, water becomes dirty, its features that made it useful are lost, and it is defined as wastewater. Consequently, the increasing volume of wastewater, which is now filled with a variety of contaminants, calls for efficient treatment methods in order to protect the water cycle and guarantee environmental sustainability.

There as on for the management of this wastewater, the inability to minimize this negativity on the water cycle, the effect of increasing population, urbanization, and industrialization has become a very critical issue in terms of environmental sustainability. The treatment of wastewater plays an important role in reducing pollutants and maintaining the quality of water resources^4^. However, the purification of new types of pollutants such as pharmaceuticals and personal care products (PPCPs), disinfection byproducts (DBPs), and polyfluoroalkyl substances (PFAS) has become more compelling^5,6^. The treatment of wastewater is not only environmentally important, but also socially and economically. In developing countries, water is polluted as a result of industrial, commercial, domestic, and agricultural activities, causing a rapid decrease in resources, thus increasing water access problems^7^. The treatment of wastewater is of great importance in terms of the protection of water resources and the realization of sustainable development goals. However, conventional wastewater treatment processes are limited in terms of sustainability due to excess energy consumption, chemical use requirement, and disposal of the sludge formed^8^. While traditional wastewater treatment methods are essential, they often struggle with delayed parameter detection, high energy use, and limited adaptability. Artificial intelligence (AI) complements these methods by enabling real-time monitoring, early anomaly detection, and predictive modeling, thereby enhancing efficiency, reducing costs, and supporting more responsive and sustainable treatment operations. In recent years, AI-based practices put forward in terms of increasing the efficiency and optimization of wastewater treatment processes have provided promising approaches^9^. These AI applications offer significant opportunities for sustainability and energy efficiency by enhancing the operating processes of wastewater treatment plants (WWTP)^10^.

AI and Machine Learning (ML) allow more efficient management of data analysis and forecasting models^11^ and wastewater treatment processes. These technological innovations are used for analyzing, making estimates, and energy optimization, beyond traditional treatment methods^12,13^. AI-based systems can monitor the biological and chemical parameters of wastewater, to predict the quality of discharge and to offer operating suggestions^14^. For example, the input and output values of parameters such as biochemical oxygen demand (BOD) and chemical oxygen demand (COD) are important indicators that provide information about the performance of WWTP^15^. The accurate and rapid estimation of these parameters plays a critical role in optimizing treatment processes and reducing operating costs. The detection of the values of these parameters after the entrance of the facility with traditional methods takes some time and does not allow the necessary interventions to be made on time. ML can learn the nonlinear relationships of these parameters and the factors affecting these parameters, making precise and reliable estimates^9,16^. In recent years, research has contributed to the development of processes that are optimized in a way that provides more efficient chemical use of AI and ML in the treatment of wastewater^16,17^. In addition, with these technologies, the existing capacity of WWTP can be increased, and a more sustainable business strategy can be adopted. These nonlinear analysis capabilities provided by AI offer an effective solution for estimating methods without entering the deep calculations of the biological and chemical kinetics of complex treatment plants^18,19^.

AI and ML are used at different stages in WWTP. These are: data collection, data-based modeling, estimation, and optimization stages. In facilities, firstly, data on the chemical and biological components of wastewater and the environmental conditions of the facility are collected^18^. A large portion of this data is continuously obtained through IoT devices and sensors, which is monitored and recorded^20^. Because IoT provides real-time monitoring and control of the system, it allows SCADA operators to detect equipment failures instantly. IoT devices that continuously monitor parameters such as plant inlet flow rates, return cycle rates, valve opening rates in the plant, flow rate, pH levels, and dissolved oxygen obtain a large portion of the data that is the basis of artificial intelligence use. Apart from these data, there are also experimental data such as COD, BOD, TN, TP, MLSSetc created in the laboratory in the facilities. AI models process all these data used in wastewater treatment. For example, algorithms such as support vector machines (SVM) and artificial neural networks (ANN) are often used for prediction and optimization processes^21^. These and similar AI algorithms can predict discharge quality and treatment costs^22^. In summary, AI applications have great potential in improving wastewater treatment processes. It plays an important role in predicting the quality of wastewater, optimizing treatment processes, and achieving sustainability goals. In the future, wider application of artificial intelligence will lead to significant improvements in reducing environmental impacts and protecting water resources by making treatment plants smarter^23^. The following are the main contributions of this study:

- In order to determine the most important influent parameters influencing effluent quality, this study methodically assesses the SelectKBest and Mutual Information (MI) RFE Random Forest feature selection techniques. The results demonstrate the importance of effluent VSS in predicting wastewater treatment performance, which facilitates more targeted monitoring and optimization.
- For the purpose of predicting COD, BOD, TSS, Total Nitrogen, and Total Phosphorus, we present a comparison between ensemble learning models (XGBoost, Random Forest, Gradient Boosting, and LightGBM) and more conventional models, such as Decision Trees.
- This study provides WWTPs with a data-driven strategy to enhance operational efficiency and regulatory compliance by demonstrating the effectiveness of ML in predicting effluent quality monitoring.

## Literature review

AI-based modeling has great potential in environmental engineering applications, particularly in complex systems such as WWTP. The most important advantage of AI is its ability to effectively model nonlinear and complex systems, even when detailed data on the functioning of real physical systems is lacking. This feature provides a significant advantage in analyzing high-dimensional and real-time data in environmental engineering. The data-driven modeling approach of AI has become popular in recent years because it is not fully coupled to the dynamics of the real system^24^. Moreover, AI stands out for its ability to accurately analyze noisy and incomplete datasets^25^. In the field of environmental engineering, AI utilizes ML and deep learning (DL) techniques to estimate parameters such as discharge quality, reuse potential, and energy consumption in wastewater and gas treatment systems^26,27^. Such applications contribute to more efficient management of water resources by making reliable predictions in environmental decision support systems. For example,^28^ used techniques such as Gene Expression Programming, Evolutionary Polynomial Regression and Model Tree stop predict future concentrations of parameters such as BOD, dissolved oxygen (DO), and COD in rivers^28^. AI tools, especially ANN, deep learning, fuzzy logic, and genetical algorithms (GA), are highly successful in modeling complex systems and making reliable predictions. These technologies are widely used in the continuous monitoring of water and air quality indices^29^. In particular, ANNs are often preferred for predicting quality in treatment processes based on direct data. In addition, ANNs provide more accurate results by modeling complex relationships between variables in environmental engineering problems.

AI applications in WWTPs aim to increase system efficiency through big data analysis, optimization, and prediction techniques. For example,^30^ achieved high accuracy rates using ANNs to predict BOD in WWTPs.^31^ combined SVM and optimization techniques to achieve high accuracy in fault diagnosis. In addition to increasing efficiency in wastewater treatment systems, AI also helps to reduce environmental impacts. For example,^32^. predicted performance with high accuracy using ANNs at the EL-AGAMY WWTP in Egypt^32^, similarly,^33^. In recent years, AI-based modeling techniques have aimed not only to increase efficiency but also to reduce environmental impacts. Wang et al. developed a successful model for phosphorus removal in a WWTP in Sweden using the XGBoost algorithm with an $\hbox {R}^2$ value of 0.88^34^. Such developments are an important step towards increasing the sustainability of WWTPs. AI-based control techniques also play a critical role in saving energy and optimizing plant performance.^15^ successfully applied ML models for COD prediction. Yu et al. utilized Transfer Learning and long short-term memory (LSTM) networks to predict wastewater COD and reduce energy consumption^35^. The majority of energy consumption in WWTPs is caused by the blowers that supply air to the system.^36^ proposed a dynamic ML model to optimize the blower operation in the aeration tank. This clearly demonstrated the importance of AI in controlling energy consumption^36^. The potential of AI in WWTPs is increasing day by day.^37^ predicted ammonia and nitrate concentrations using LSTM, and^38^ dynamically regulated DO concentration using Reinforcement Learning (RL). These studies prove the effectiveness of AI in control processes.

Despite the growing body of literature emphasizing the potential of AI models particularly deep learning and neural networks-in modeling complex, nonlinear systems within wastewater treatment plants (WWTPs)^26,30,32^, a notable gap remains in systematically comparing these AI approaches with traditional statistical models such as linear regression (LR). While AI techniques like XGBoost, LSTM, and ANNs have demonstrated high predictive accuracy for key wastewater indicators such as BOD, COD, and phosphorus^30,34,35^, they are often criticized for their “black-box” nature and higher computational requirements, which can limit interpretability and practical deployment in many facilities^31,36^. This study addresses that gap by offering a structured comparison between ensemble learning models (XGBoost, Random Forest, Gradient Boosting, LightGBM) and simpler models such as Decision Trees, thus bridging the divide between performance and explainability with the feature selection techniques.

## Methodology

**Fig. 1:**
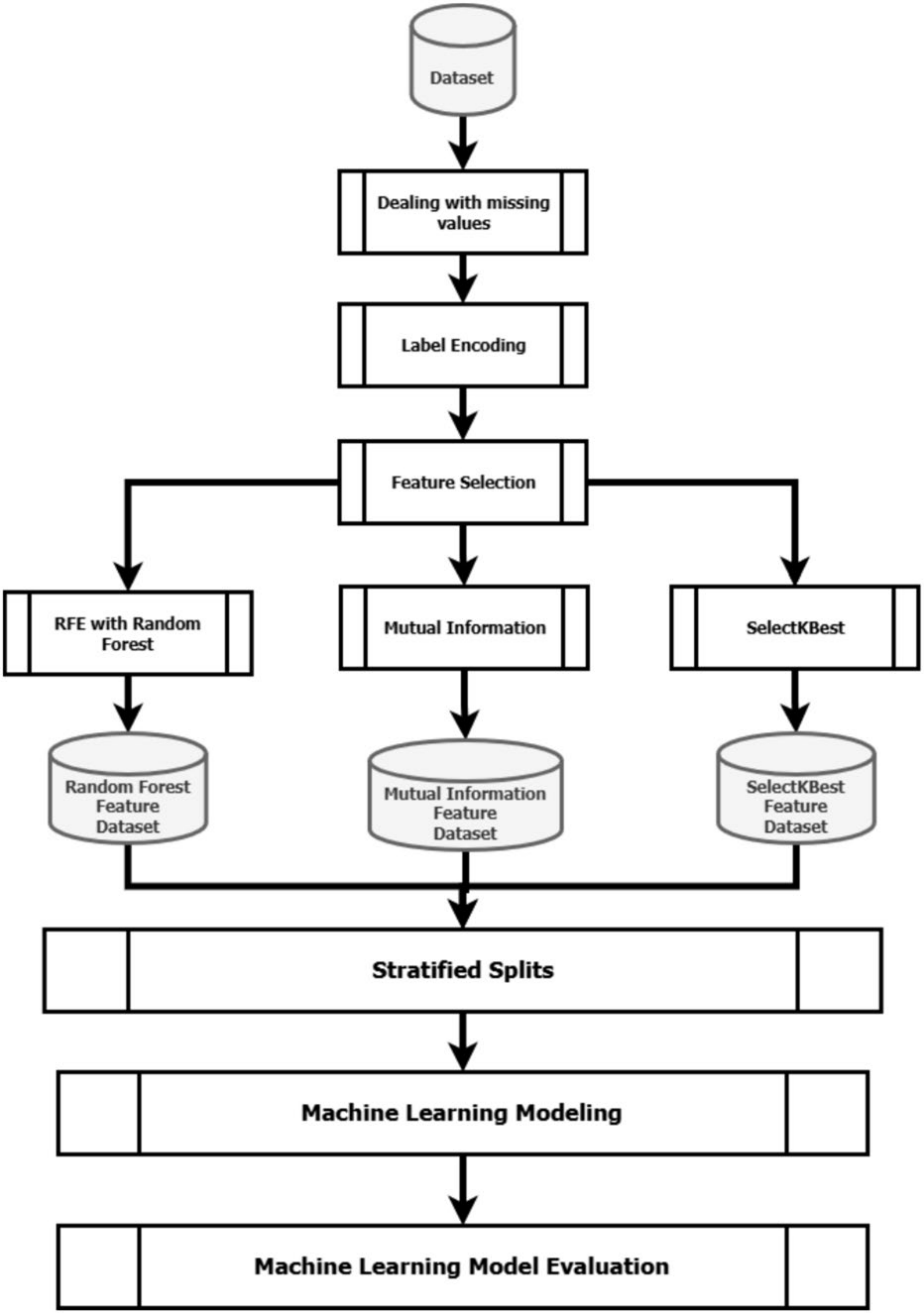
Research methodology flow chart for the study.

In this study, ML models are developed to predict potential disturbances in a WWTP’s discharge. The system consists of 6 layers: Dealing with missing values, Label Encoding, Feature Selection, Stratified Splits, ML Modeling, and ML Model Evaluation. The following section discusses each layer in detail. The study’s methodology is represented in Fig. 1.

### Dataset

The dataset includes information about ambient conditions, facilities, laboratories, and vital facilities. The data collection period runs from January 1, 2022, to December 8, 2024. The data is collected on a daily basis. The dataset has 1075 rows. It includes 65 features and 6 target variables. The target variables are COD, BOD, Total Suspended Solids (TSS), Effluent Total Nitrogen, and Effluent Total Phosphorus. Table 1 shows how features of datasets are divided into categories. Four major groups of features related to wastewater treatment analysis are presented in Table 1. Important infrastructure elements like diffusers and aeration tanks fall under the Important Facility Parts category. Indicators of influent and effluent water quality, such as organic and chemical oxygen demand, nitrogen, phosphorus levels, and other vital parameters, are included in laboratory data. Information about the facility includes operational metrics such as energy consumption, chemical usage, sludge management, and flow rates. The last section, Environmental Conditions, discusses outside variables that can affect the effectiveness of wastewater treatment, including temperature, humidity, and weather. The dataset was collected from lab tests, plant logs, sensor readings, and local weather stations. Standard procedures guaranteed data accuracy, and preprocessing steps handled any missing or inconsistent values.

**Table 1:** Feature and their categories.

Category	Features
Important facility parts	Active aeration tank amount, active final settling tank amount, active diffuser amount
Laboratory data	Influent COD, influent dissolved COD SCOD IAT, effluent dissolved COD SCOD inert EST, influent BOD influent TSS, influent VSS, effluent VSS, influent total nitrogen, influent ammonia nitrogen, effluent ammonia nitrogen, influent nitrate nitrogen, effluent nitrate nitrogen, influent total phosphorus, polymer concentration, DSV (diluted sludge volume), MLSS (mixed liquor suspended solids), MLVSS (mixed liquor volatile suspended solids), tank pH, influent alkalinity, reactor temperature, $O_2$ concentration, salinity
Facility data	Process flow rate, return sludge pump flow rate, internal recirculation, excess sludge pump flow rate, sludge pump flow rate to digester—primary sludge, sludge pump flow rate to digester after thickening—secondary sludge, sludge quantity, DS content of sludge, dry product quantity, biogas quantity, polymer pump flow rate, methanol usage, iron usage, hourly average blower flow rate, hourly average blower energy consumption, daily total energy consumption
Environmental conditions	Weather condition, air temperature, relative humidity

### Dealing with missing values

The quality of the data affects the ML models’ dependability and quality. A major problem with missing values is that they can lead to bias and reduced model accuracy if handled incorrectly. The main reason for missing values in the dataset is usually sensor failure, which is a common occurrence for the use case in this study. This study makes use of mean imputation, a popular statistical method that substitutes the average of the observed values for a feature for missing values in numerical characteristics. Mean imputation was chosen for its simplicity and efficiency in preserving the overall distribution of numerical features. It performs well when missingness is random and the variable’s distribution is approximately normal. Mean imputation is done using equation 1

1 $$\begin{aligned} x_i = {\left\{ \begin{array}{ll} x_i, & \text {if } x_i \text { is not missing} \\ \frac{1}{N} \sum \limits _{j=1}^{N} x_j, & \text {if } x_i \text { is missing} \end{array}\right. } \end{aligned}$$

In Eq. 1$x_i$ is the imputed value, *N* is the population, and $x_j$ is the value of the feature at an instance.

### Label encoding

After missing values have been removed, label encoding, a method for transforming categorical values into numerical representations, is carried out. This transformation is necessary to utilize ML models that require numerical input. To ensure that the model can efficiently interpret categorical data, label encoding assigns a distinct integer to each category within a feature. Equation 2 is its mathematical representation for label encoding.

2 $$\begin{aligned} LE(x) = i, \quad \text {where } x \in X, \quad i = {0, 1, 2, \ldots , n-1} \end{aligned}$$

### Feature engineering

To improve model performance, feature engineering is essential for choosing the most important features from a dataset. A variety of feature selection strategies are used to lower computational complexity and increase predictive accuracy. SelectKBest, MI-based selection, and Recursive Feature Elimination (RFE) using Random Forest are selected techniques. SelectKBest, Mutual Information, and RFE are chosen for their complementary strengths: SelectKBest offers fast univariate filtering, MI captures both linear and non-linear relationships, and RFE provides model-based feature ranking. Together, they balance interpretability, computational efficiency, and robustness in handling complex environmental data.

#### SelectKBest

The SelectKBest is a prominent feature selection approach that is frequently utilized in data preprocessing. Its goal is to reduce the dimension of the feature by identifying the most relevant features for a certain target. The ’K’ in SelectKBest refers to the total number of features to be selected. In our investigation, we chose 10 features with the aim of reducing the dimension of the feature set. After implementing feature selection, the total number of features decreased significantly from 65 to 10. Equation 3 represents SelectKBest mathematically.

3 $$\begin{aligned} S_k = \arg \max _{S, |S|=k} \sum _{f \in S} I(f, Y) \end{aligned}$$

In eqaution 3$S_k$ represents the selected subset of $k$ features, $I(f, Y)$ denotes the scoring function that measures the relevance of feature $f$ with respect to the target variable $Y$, and $|S|=k$ ensures that exactly $k$ features are chosen.

#### Mutual information

MI measures the dependence of the target variable on the input features. MI is a robust feature selection technique that, in contrast to correlation-based methods, captures both linear and non-linear relationships. Features with higher mutual information scores are given preference during selection because they have a substantial impact on model performance. The reduction of one variable’s uncertainty in light of another’s knowledge is known as MI. Mathematically, mutual information between a feature $X$ and the target variable $Y$ is given by eqaution 4

4 $$\begin{aligned} I(X; Y) = \sum _{x \in X} \sum _{y \in Y} p(x, y) \log \left( \frac{p(x, y)}{p(x) p(y)} \right) \end{aligned}$$

In Eq. 4 represents the mutual information between feature $X$ and target $Y$, $p(x, y)$ is the joint probability distribution of $X$ and $Y$, and $p(x)$ and $p(y)$ are the marginal probability distributions of $X$ and $Y$, respectively.

#### Recursive feature elimination (RFE) using random forest

To enhance model performance, a feature selection method called RFE iteratively eliminates the least significant features. Fitting a model, prioritizing features, and recursively removing the least important features until the required number of features are left is how it operates. RFE uses the built-in feature importance scores of Random Forest, an ensemble learning technique based on decision trees, to direct the selection process. Feature importance in Random Forest is typically computed using the Gini Importance, which is represented by Eq. 5

5 $$\begin{aligned} G(X_i) = \sum _{t \in T} p(t) \cdot \left( G_{\text {before}}(t) - G_{\text {after}}(t) \right) \end{aligned}$$

In Eq. 5$G(X_i)$ represents the importance of feature $X_i$, $T$ is the set of decision tree nodes where $X_i$ is used for splitting, $p(t)$ is the proportion of samples reaching node $t$, and $G_{\text {before}}(t)$ and $G_{\text {after}}(t)$ are the Gini impurity measures before and after the split at node $t$, respectively. Algorithm 1 shows the entire step-by-step process of RFE using Random Forest.

**Algorithm 1:**
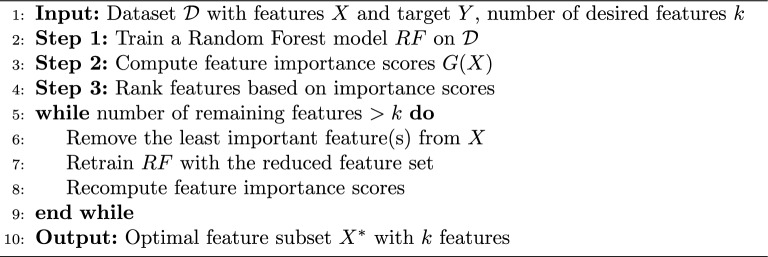
Recursive Feature Elimination (RFE) using Random Forest

### Stratified split

Stratified splitting is an ML technique that ensures the target variable distribution in the training and testing sets matches that of the original dataset. In this study, we experimented with various train-test splits, such as 80-20, 70-30, 60-40, and 50-50 proportions, to assess model performance. The results presented in this paper are for the 80-20 split only. Stratified splitting can be expressed mathematically as follows.

6 $$\begin{aligned} P(Y_{train} \in B_i) \approx P(Y_{test} \in B_i) \approx P(Y \in B_i), \end{aligned}$$

In Eq. 6$B_i$ represents a bin that groups target values into similar ranges, ensuring that both the training and testing sets maintain a similar distribution of target values.

### Machine learning modeling

Following preprocessing, ML modeling is applied to the data in this step, using the training proportion of the data extracted in the last step. The chosen algorithms include Decision Tree, Random Forest, Gradient Boosting, XGBoost, and LightGBM. Each algorithm is discussed in the following sections.

#### Decision tree

Decision Tree Regression is a robust and user-friendly ML method for predicting continuous values. It is a non-linear regression technique that can handle complex datasets with intricate patterns, in contrast to traditional linear regression, which assumes a straight-line relationship between input features and the target variable. It is adaptable and straightforward to understand because it makes predictions using a tree-like model. Decision trees use decision rules derived from the input features to divide the data into smaller subsets, producing accurate predictions. Every split aims to lower the prediction error for the target variable. The algorithm predicts a continuous value, typically the average of the goal values in that node, at each leaf node of the tree.

#### Random forest

A random subset of the data is used to train each of the many decision trees produced by Random Forest Regression. The process starts with Bootstrap sampling, which generates distinct training datasets for every tree by choosing random rows of data with replacement. To ensure model diversity, we then perform feature sampling, which involves creating each tree using a random selection of attributes. After training, each tree forecasts, and the average of all the individual tree predictions-a process called aggregation-is the final prediction for regression tasks.

#### Gradient boosting

Gradient boosting is an effective ML method that builds models step-by-step and incrementally. Through the process of training successive models, the overall prediction accuracy is gradually increased as the models learn to correct the mistakes of the previous models. Gradient boosting works on the premise of building a strong ensemble model by combining several weak predictive models, typically decision trees. Algorithm 2 shows the step-by-step process of gradient boosting. Making a foundational model is the first step, and this could be as easy as creating a decision tree. Every instance in the dataset has its initial predictions produced by this model. The computation of each prediction’s residuals, or errors, is the second stage. Variations between expected and actual values are represented by residuals. The errors made by the previous trees are essentially fixed in the third step, where each new tree in the sequence is trained to predict the residuals of the previous tree. In step four, the model’s parameters are changed in order to minimize the loss function. To accomplish this, new trees are fitted to the loss function’s negative gradient, allowing for gradual boosting. Lastly, the weighted predictions from each tree are added together to produce the overall prediction. Usually, the learning rate determines the weights, which regulate the rate of learning.

**Algorithm 2:**
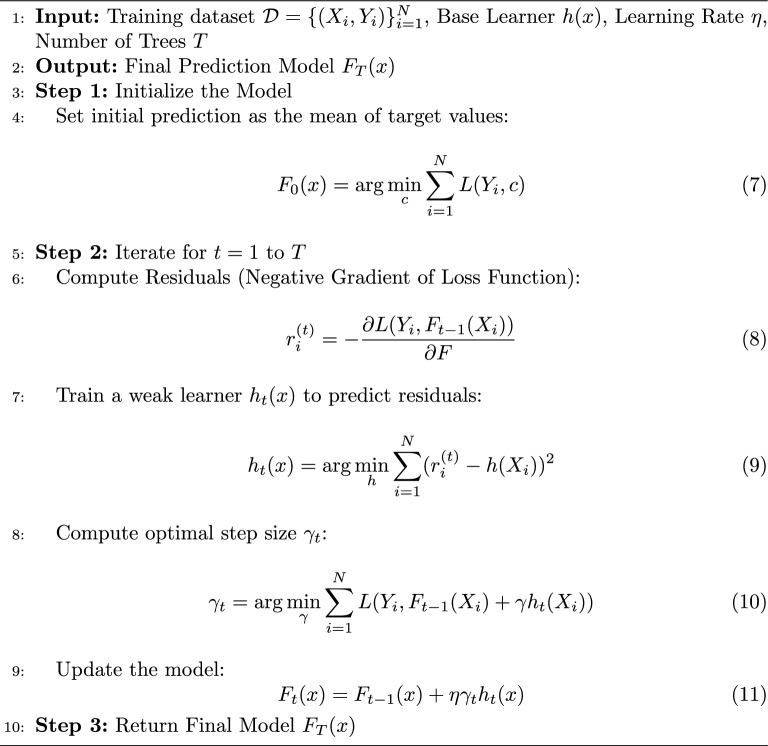
Gradient Boosting Regression

#### XGBoost

XGBoost is one of the most effective methods for creating supervised regression models. Understanding the objective function and base learners of XGBoost allows one to deduce the validity of this claim. A regularization term and a loss function are included in the objective function, which describes how actual values differ from predicted values, i.e., the degree to which the model’s output differs from the actual values. The most popular loss functions in XGBoost are reg:linear for regression problems. XGBoost is an ensemble learning technique that entails training and merging separate models (referred to as base learners) to produce a single prediction. In order for bad predictions to cancel out and better predictions to add up to final good predictions, XGBoost anticipates that the base learners will be uniformly bad at the remainder.

#### LightGBM

Microsoft created LightGBM, a distributed, open-source, high-performance gradient boosting framework, designed with accuracy, scalability, and efficiency in mind. Its foundation is based on decision trees, which aim to enhance model effectiveness and reduce memory consumption. In order to maximize memory usage and training time, it uses a number of innovative techniques, such as Gradient-based One-Side Sampling (GOSS), which retains instances with large gradients during training. Histogram-based algorithms are also used by LightGBM for effective tree construction. In addition to optimizations like leaf-wise tree growth and effective data storage formats, these strategies help LightGBM be more efficient and provide it with a competitive advantage over other gradient boosting frameworks.

### Machine learning model evaluation

This section discusses the evaluation metrics used in this study. These models are evaluated against the test set from the dataset. The metrics employed in this study are the root mean squared error (RMSE), mean absolute error (MAE), and coefficient of determination ($R^2$).

#### Root mean square error (RMSE)

The standard deviation (prediction errors) of the residuals, also known as RMSE, is a measure of how dispersed these residuals are. Residuals, on the other hand, measure how far away the regression line’s data points are. It provides information about the degree of concentration of the data around the line of best fit. To validate experimental results, regression analysis frequently uses RMSE. Equation 7 shows how RMSE is calculated.

7 $$\begin{aligned} \text {RMSE} = \sqrt{\frac{1}{n} \sum _{i=1}^{n} (y_i - \hat{y}_i)^2} \end{aligned}$$

In Eq. 7 n is the number of samples, $y_i$ is the actual value, and $\hat{y}_i$ is the predicted value.

#### Mean absolute error (MAE)

The accuracy of regression models can be assessed using the straightforward but effective MAE metric. It calculates the mean absolute difference between the target values and the predicted values. In contrast to other metrics, MAE assigns equal weight to all errors, regardless of their direction, because it does not square the errors. When you wish to comprehend the extent of errors without taking into account whether they are overestimations or underestimations, this feature makes MAE especially helpful. Equation 8 shows the mathematical formula for MAE

8 $$\begin{aligned} \text {MAE} = \frac{1}{n} \sum _{i=1}^{n} \left| y_i - \hat{y}_i \right| \end{aligned}$$

In Eq. 8 n is the number of samples, $y_i$ is the actual value, and $\hat{y}_i$ is the predicted value.

#### Coefficient of determination ($R^2$)

The coefficient of determination is a percentage; it offers a viewpoint on how several data points might coincide with the line produced by the reversal equation. When plotting the data points and the line consumed, the higher the coefficient, the higher the percentage of the fact line flows through. The percentage of the dependent variable’s variance that can be predicted from the independent variable is perhaps another way to express the coefficient of determination. Within the regression line, 80% of the points will fall if the coefficient is 0.80. The sign of a better fit between the statements is a longer coefficient. Equation 9 represents the coefficient of determination ($R^2$) mathematically.

9 $$\begin{aligned} R^2 = 1 - \frac{\sum _{i=1}^{n} (y_i - \hat{y}_i)^2}{\sum _{i=1}^{n} (y_i - \bar{y})^2} \end{aligned}$$

In Eq. 9 n is the number of samples, $y_i$ is the actual value, $\hat{y}_i$ is the predicted value, $\bar{y}$ and is the mean of the observed values.

## Results

In this section, we present the empirical findings from several ML models that predicted COD, BOD, TSS, Effluent Total Nitrogen, and Effluent Total Phosphorus in wastewater from WWTP. The evaluation metrics used for this task were Mean Square Error (MSE), MAE, and R-squared ($R^2$).

### Experimental settings

The studies were conducted using Google Colab, a cloud-based Jupyter Notebook environment. The Python implementation utilized key libraries, including Matplotlib for data visualization, NumPy for numerical calculations, Pandas for data manipulation and preprocessing, and Scikit-learn for ML modeling and evaluation. In this study, no hyperparameter optimization was applied, so the default hyperparameters were used for all algorithms. The computer environment had 16GB of RAM without GPU acceleration. CPUs were used for both model evaluation and training.

### Empirical results

**Fig. 2:**
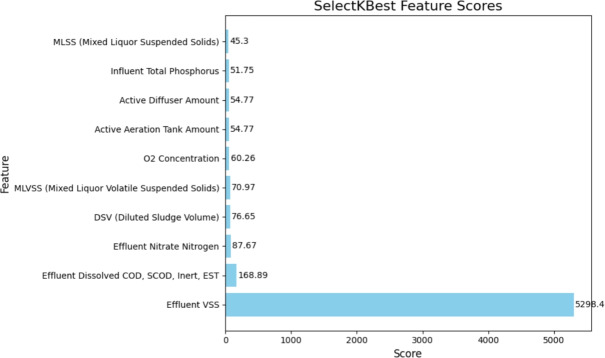
Top 10 feature score for KSelect method.

The feature importance scores derived using the SelectKBest method are shown in the bar chart in Fig. 2. Based on the graph, Effluent VSS is the most important feature based on this selection method, as evidenced by its highest score 5298.4. With a significantly lower score, 168.89, than Effluent VSS, the second most important feature-Effluent Dissolved COD, SCOD, Inert, EST-shows a sharp decline in importance. With progressively declining scores, the other features come next, with Mixed Liquor Suspended Solids (MLSS) scoring the lowest of the top 10 45.3.

The Table 2 shows the MAE and MSE for several ML models using the SelectKBest feature selection method. Based on the Table 2 XGBoost has the lowest MSE (119.24) for predicting effluent COD, Random Forest has the lowest MAE (6.02), and Decision Tree performs the worst. Random Forest performs the best in predicting effluent BOD, with the lowest MAE (1.62) and MSE (6.08), while Decision Tree performs the worst. Gradient Boosting is the best model for predicting effluent TSS because it has the lowest MSE (56.45) and MAE (3.73), whereas LightGBM has the highest error. Gradient Boosting performs better than other models in the prediction of effluent total nitrogen, with the lowest MAE (0.67) and MSE (1.12), whereas Decision Tree has the highest errors. Finally, LightGBM achieves the lowest MSE (0.21) for predicting Effluent Total Phosphorus, while Decision Tree performs the worst with the highest errors.

**Table 2:** Performance comparison of machine learning models for predicting effluent parameters using the SelectKBest feature selection method.

SelectKBest			
Target	Model	MAE	MSE
Effluent COD	Random Forest	6.019023	127.8496
Effluent COD	Gradient Boosting	6.063454	135.7005
Effluent COD	XGBoost	6.251666	119.2433
Effluent COD	LightGBM	6.75571	157.9351
Effluent COD	Decision Tree	9.039535	330.1942
Effluent BOD	Random Forest	1.623498	6.079346
Effluent BOD	Gradient Boosting	1.645596	6.076093
Effluent BOD	XGBoost	1.686399	6.331241
Effluent BOD	LightGBM	1.768605	7.552577
Effluent BOD	Decision Tree	2.295814	14.89609
Effluent TSS	Random Forest	4.187265	87.94894
Effluent TSS	Gradient Boosting	3.732114	56.45357
Effluent TSS	XGBoost	4.629108	131.9035
Effluent TSS	LightGBM	6.028417	312.5321
Effluent TSS	Decision Tree	5.166512	127.2104
Effluent total nitrogen	Gradient Boosting	0.673567	1.116201
Effluent total nitrogen	XGBoost	0.746523	1.954209
Effluent total nitrogen	LightGBM	0.772575	1.790108
Effluent total nitrogen	Decision Tree	1.083744	5.730707
Effluent total phosphorus	Random Forest	0.227669	0.241111
Effluent total phosphorus	Gradient Boosting	0.223368	0.239269
Effluent total phosphorus	XGBoost	0.224297	0.227791
Effluent total phosphorus	LightGBM	0.230975	0.210283
Effluent total phosphorus	Decision Tree	0.330349	0.465003

**Fig. 3:**
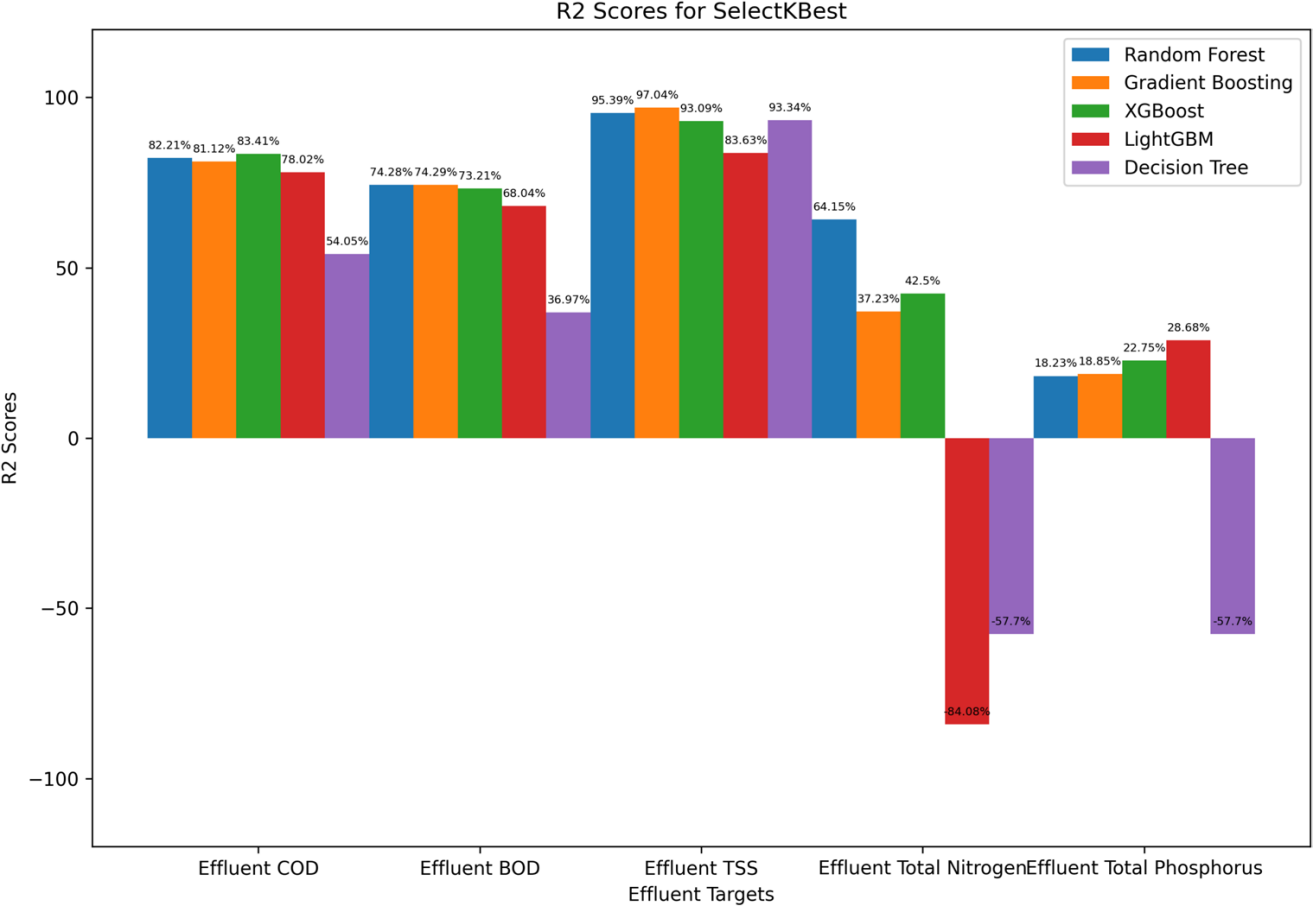
KSelect based performance evaluation ($R^2$) of machine learning algorithm.

The bar chart in Fig. 3 presents the performance evaluation of various ML models using the SelectKBest feature selection method, measured by $\hbox {R}^2$ score for different effluent parameters. For Effluent COD, XGBoost (83.41%) outperforms Random Forest (82.21%) and Gradient Boosting (81.12%), while Decision Tree (54.05%) performs the worst. For Effluent BOD, Gradient Boosting (74.29%) and Random Forest (74.28%) show the best results, whereas Decision Tree (36.97%) is the least effective. For Effluent TSS, Gradient Boosting (97.04%) achieves the highest performance, followed by Random Forest (95.39%), with LightGBM (83.63%) trailing behind. In Effluent Total Nitrogen, Gradient Boosting (64.15%) outperforms other models, while Decision Tree (-84.08%) performs significantly worse. For Effluent Total Phosphorus, LightGBM (28.68%) leads, whereas Decision Tree (-57.70%) fails to capture meaningful patterns.

**Fig. 4:**
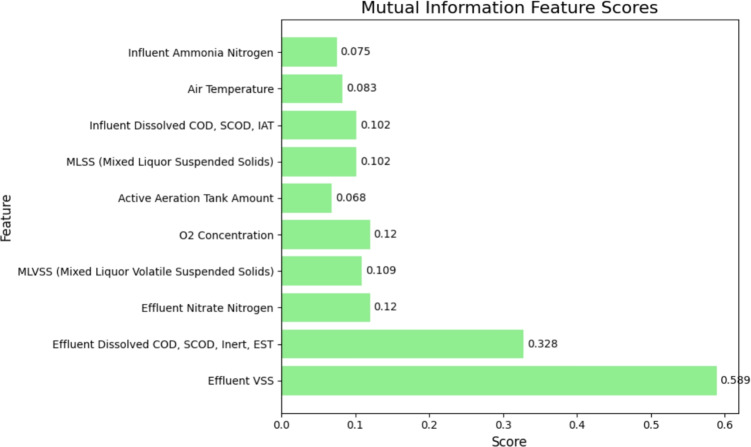
Top 10 feature score for mutual information method.

Figure 4 illustrates the top 10 features selected using the Mutual Information method, ranked based on their respective scores. Effluent VSS (0.589) exhibits the highest mutual information score, indicating its strong relevance in predicting the target variable. This is followed by Effluent Dissolved COD, SCOD, Inert, EST (0.328), and Effluent Nitrate Nitrogen (0.12). Other important features include MLVSS (0.109), $O_{2}$ Concentration (0.12), and MLSS (0.102), which contribute moderately to the model’s predictive power. Air Temperature (0.083) and Influent Ammonia Nitrogen (0.075) show relatively lower importance but still hold valuable information.

A variety of ML models trained with features chosen through the MI method are shown in Table 3. Decision Tree performed the worst for Effluent COD, with MAE of 10.41 and MSE of 444.50. At the same time, Random Forest obtained the lowest MAE (6.2485) and MSE (156.35). Likewise, for Effluent BOD, XGBoost performed the best (MAE: 1.5898, MSE: 4.8127), while Decision Tree had the highest errors. The MSE (47.23) for Effluent TSS was the lowest for Gradient Boosting, and the MSE (307.05) for LightGBM. While Decision Tree had the highest error values, LightGBM had the lowest MSE (2.44) for Effluent Total Nitrogen. Finally, for Effluent Total Phosphorus, Decision Tree performed the worst, and XGBoost had the lowest MSE (0.2519). In terms of predictive accuracy, these findings demonstrate that ensemble models such as Random Forest, XGBoost, and Gradient Boosting typically perform better than single-tree models like Decision Tree.

**Table 3:** Performance comparison of machine learning models for predicting effluent parameters using the mutual information feature selection method.

Mutual information			
Target	Model	MAE	MSE
Effluent COD	Random Forest	6.248535	156.3548
Effluent COD	Gradient Boosting	6.275448	165.9364
Effluent COD	XGBoost	6.474302	149.3885
Effluent COD	LightGBM	6.838371	168.7026
Effluent COD	Decision Tree	10.41163	444.4965
Effluent BOD	Random Forest	1.610291	5.704391
Effluent BOD	Gradient Boosting	1.663382	6.274504
Effluent BOD	XGBoost	1.589808	4.812732
Effluent BOD	LightGBM	1.771592	7.35923
Effluent BOD	Decision Tree	2.377209	14.0647
Effluent TSS	Random Forest	3.972572	58.8857
Effluent TSS	Gradient Boosting	3.667118	47.23402
Effluent TSS	XGBoost	4.624556	136.5479
Effluent TSS	LightGBM	6.148256	307.0467
Effluent TSS	Decision Tree	4.970698	90.35949
Effluent total nitrogen	Random Forest	0.938949	2.548645
Effluent total nitrogen	Gradient Boosting	0.915302	2.552992
Effluent total nitrogen	XGBoost	0.925648	2.608186
Effluent total nitrogen	LightGBM	0.902001	2.441467
Effluent total nitrogen	Decision Tree	1.230209	5.732397
Effluent total phosphorus	Random Forest	0.236964	0.276596
Effluent total phosphorus	Gradient Boosting	0.249694	0.347067
Effluent total phosphorus	XGBoost	0.235134	0.251872
Effluent total phosphorus	LightGBM	0.244911	0.239556
Effluent total phosphorus	Decision Tree	0.334651	0.539802

**Fig. 5:**
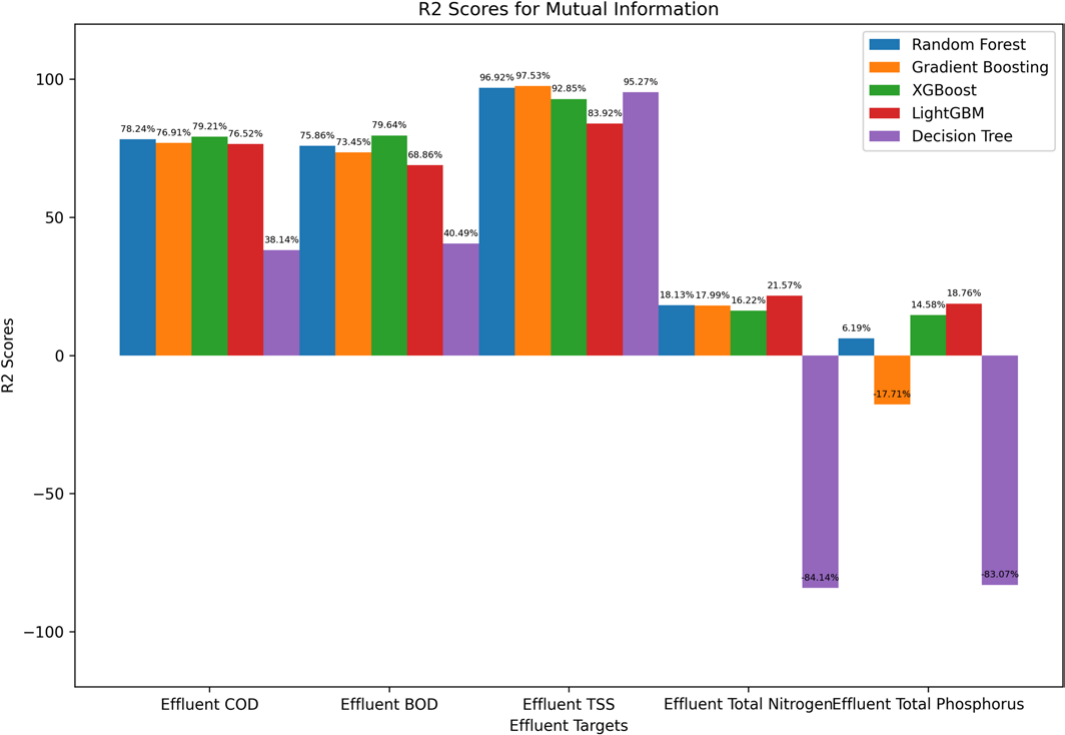
Mutual information-based performance evaluation ($R^2$) of machine learning algorithm.

The $R^2$ scores of several ML models trained with features chosen using the MI method are shown in Fig. 5. With a $R^2$ of 79.21%, XGBoost outperformed Effluent COD, closely followed by Random Forest (78.24%), while Decision Tree trailed far behind at 38.14%. Effluent BOD shows a similar pattern, with XGBoost leading with 79.64% and Decision Tree scoring only 40.49%. Decision Tree performed similarly well at 95.27% for Effluent TSS, while Gradient Boosting performed the best with 97.53%, followed by Random Forest (96.92%). Performance was generally poor for Effluent Total Nitrogen, with LightGBM (21.57%) outperforming the others and Decision Tree (-84.14%) demonstrating a significant negative correlation. Similar trends were seen for Effluent Total Phosphorus, with Decision Tree (-83.07%) performing the worst and LightGBM (18.76%) performing the best. These findings show that ensemble models consistently outperform Decision Tree models in terms of predictive accuracy across a range of effluent parameters, especially XGBoost, Gradient Boosting, and Random Forest.

**Fig. 6:**
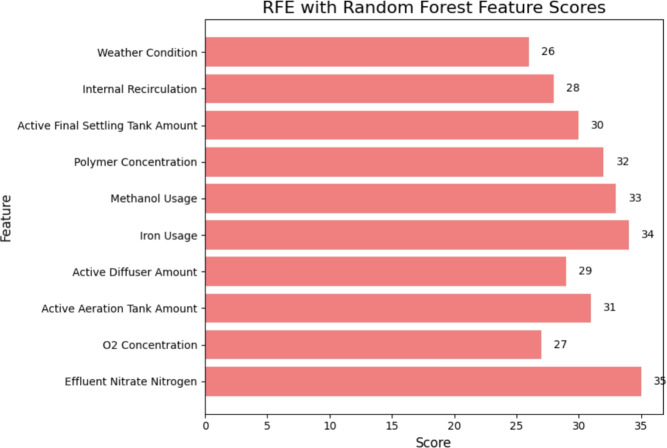
Top 10 feature score for RFE random forest method.

Using Random Forest and the RFE method, the top 10 feature scores are shown in Fig. 6. In terms of the model’s performance, Effluent Nitrate Nitrogen (35) is the most important feature, followed by Iron Usage (34) and Methanol Usage (33). In order to predict effluent quality, other important parameters are Polymer Concentration (32), Active Aeration Tank Amount (31), and Active Final Settling Tank Amount (30). Other significant factors include Active Diffuser Amount (29), Internal Recirculation (28), $O_{2}$ Concentration (27), and Weather Condition (26).

In Table 4, RFE with the Random Forest method is used to evaluate the performance of ML models. In order to predict effluent quality parameters, the MAE and MSE are described. With Effluent COD prediction displaying comparable MAE values across all models ( 14.96), Gradient Boosting and XGBoost are the models that consistently achieve competitive error metrics. Effective BOD prediction shows minimal errors, and LightGBM does marginally better in MAE (2.7912). There are slight differences in the Effluent TSS prediction results; Random Forest had the lowest MSE (1754.429). For Effluent Total Nitrogen, Random Forest has the lowest MAE (1.0692), and LightGBM has the lowest MSE (0.2678), indicating that Effluent Total Phosphorus shows few errors across models. These findings demonstrate that, although tree-based models perform well, XGBoost and Gradient Boosting offer consistent performance across a variety of effluent quality measurements.

**Table 4:** Performance comparison of machine learning models for predicting effluent parameters using the RFE random forest feature selection method.

RFE Random Forest			
Target	Model	MAE	MSE
Effluent COD	Random Forest	14.98364	648.2635
Effluent COD	Gradient Boosting	14.95776	648.7881
Effluent COD	XGBoost	14.95892	648.7051
Effluent COD	LightGBM	14.97676	649.464
Effluent COD	Decision Tree	14.95892	648.7049
Effluent BOD	Random Forest	2.795836	21.13264
Effluent BOD	Gradient Boosting	2.798389	21.14774
Effluent BOD	XGBoost	2.799759	21.14599
Effluent BOD	LightGBM	2.791269	21.14876
Effluent BOD	Decision Tree	2.799772	21.14602
Effluent TSS	Random Forest	20.84885	1754.429
Effluent TSS	Gradient Boosting	20.82707	1754.651
Effluent TSS	XGBoost	20.82904	1754.541
Effluent TSS	LightGBM	20.73614	1757.036
Effluent TSS	Decision Tree	20.82904	1754.54
Effluent Total Nitrogen	Random Forest	1.069232	3.1446
Effluent Total Nitrogen	Gradient Boosting	1.073397	3.153474
Effluent Total Nitrogen	XGBoost	1.073701	3.154087
Effluent Total Nitrogen	LightGBM	1.077928	3.167588
Effluent Total Nitrogen	Decision Tree	1.073708	3.154098
Effluent Total Phosphorus	Random Forest	0.265439	0.272837
Effluent Total Phosphorus	Gradient Boosting	0.26712	0.273593
Effluent Total Phosphorus	XGBoost	0.267285	0.273718
Effluent Total Phosphorus	LightGBM	0.262978	0.267874
Effluent Total Phosphorus	Decision Tree	0.267298	0.273719

**Fig. 7:**
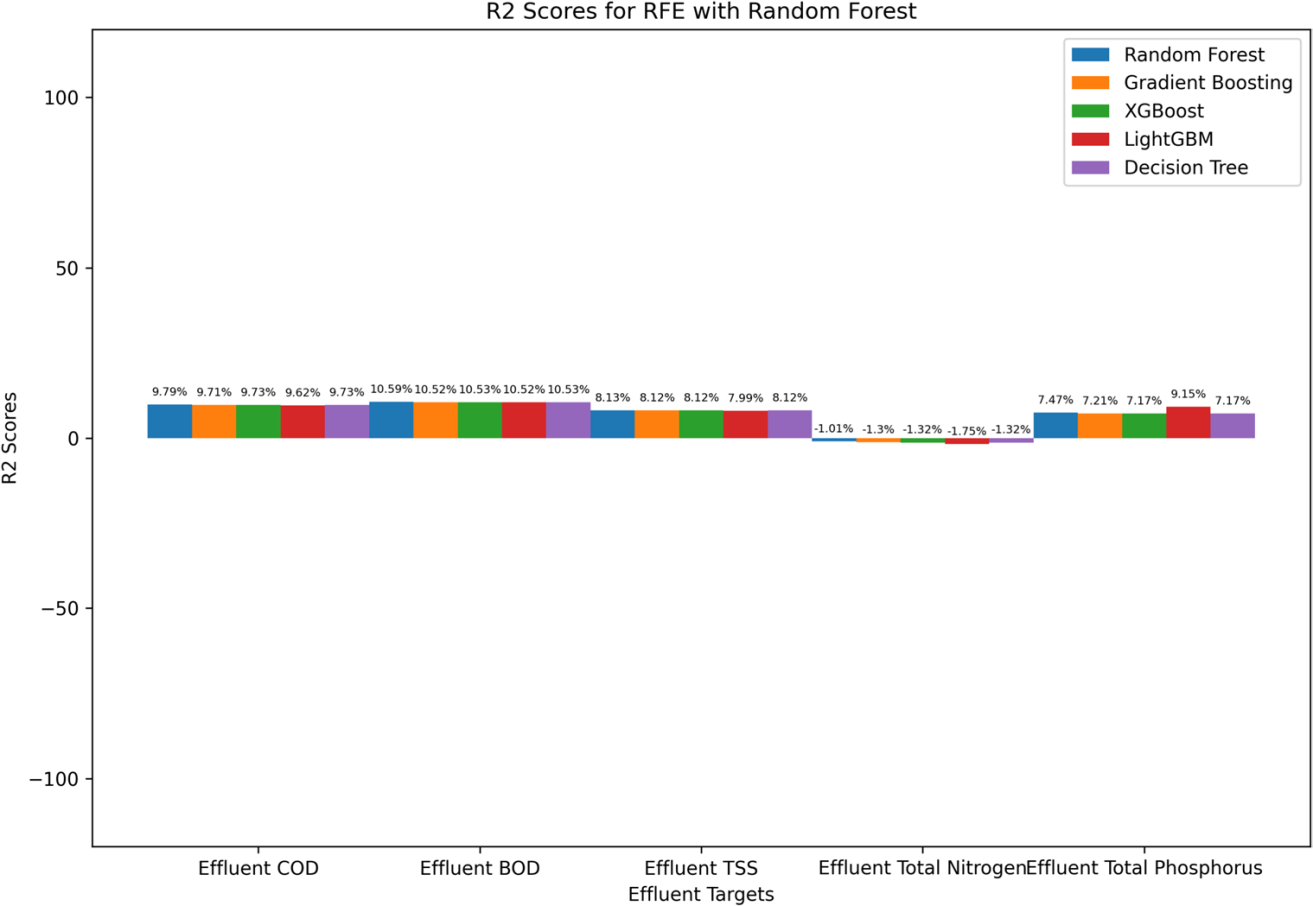
RFE random forest based performance evaluation ($R^2$) of machine learning algorithm.

Figure 7 illustrates RFE with Random Forest-based $R^2$ performance evaluation of various ML models for predicting effluent quality parameters. The results indicate that Effluent COD and BOD predictions achieve relatively higher $R^2$ values, with Random Forest yielding the highest performance (9.79% and 10.59%, respectively). Effluent TSS predictions exhibit consistent performance across models, with a slight advantage for Random Forest (8.13%). However, Effluent Total Nitrogen predictions show negative $R^2$ values, suggesting that the models struggle to explain variance, with LightGBM performing the worst (-1.75%). In contrast, Effluent Total Phosphorus prediction shows the highest $R^2$ for LightGBM (9.15%), surpassing other models. These findings highlight that while tree-based models perform well for some effluent parameters, their effectiveness varies depending on the target variable.

## Discussion and conclusion

In this study, we investigated the predictive accuracy of ML models for effluent parameters in wastewater treatment facilities, focusing on feature selection strategies to enhance model performance. The analysis revealed that Effluent VSS consistently holds the highest predictive importance when utilizing the SelectKBest and Mutual Information, RFE Random Forest methods to identify the most significant features. These findings align well with operational knowledge of wastewater treatment processes. VSS is a key indicator of the biological activity in the treatment system, particularly in the activated sludge process, where microorganisms decompose organic matter. Other important features, such as influent flow rate or sludge retention time, also correlate with system dynamics, influencing hydraulic loading and the effectiveness of biological treatment.

XGBoost outperformed Effluent BOD in terms of MAE, also XGBoost obtained the lowest MSE for Effluent COD. LightGBM produced the most accurate predictions for Effluent Total Phosphorus when using SelectKBest, while Gradient Boosting was the most successful for Effluent TSS and Total Nitrogen. On the other hand, Decision Tree consistently produced the highest errors and performed the worst. Ensemble methods such as XGBoost, Gradient Boosting, and LightGBM outperformed others due to their ability to capture complex, non-linear relationships among feature vectors. Boosting techniques reduce overfitting, making these models less sensitive to noise, which is well-suited for environmental datasets where data variability is high. In contrast, simpler models like linear regression and decision trees may have underperformed due to their limited capacity to model non-linear relationships.

Despite these encouraging findings, the study has certain limitations. Even though the dataset is extensive, it might not adequately account for operational irregularities and seasonal variations in wastewater treatment. Using feature selection techniques that only consider statistical significance may also ignore domain-specific elements that affect effluent quality.

To address the limitations, firstly, future research would incorporate operational logs from treatment plants and time series data spanning multiple seasons. Secondly, future work would adopt hybrid feature selection approaches that combine statistical methods with expert-driven criteria to better align with practical operational insights. Furthermore, future work would focus on integrating AI predictions into real-time control systems, enabling automated adjustments to operations based on predicted effluent quality, thereby improving efficiency and sustainability in wastewater management. Finally, the combination of hybrid models and deep learning techniques will be investigated using real-time sensor data to enhance prediction accuracy and facilitate spatiotemporal analysis.

The models developed in this study can support more efficient chemical dosing, reduce energy consumption in WWTP systems, and help treatment facilities maintain compliance with discharge regulations, contributing both to cost savings and environmental protection.

## Data Availability

The data supporting the findings of this study are available from the Istanbul Water and Wastewater Administration; however, restrictions apply to the availability of these data, which were used under license for the current study and so are not publicly available. Data are, however, available from the author (Faruk Dikmen, email: faruk.dikmen@std.yildiz.edu.tr) upon reasonable request and with permission of the Istanbul Water and Wastewater Administration.
